# Spatio-Temporal Variation in the Concentration of Inhalable Particulate Matter (PM_10_) in Uganda

**DOI:** 10.3390/ijerph16101752

**Published:** 2019-05-17

**Authors:** Silver Onyango, Beth Parks, Simon Anguma, Qingyu Meng

**Affiliations:** 1 Faculty of Science, Mbarara University of Science and Technology, Mbarara, Uganda; silvonya165@gmail.com; 2 Department of Physics and Astronomy, Colgate University, Hamilton, NY 13346, USA; 3 Department of Physics, Muni University, Arua, Uganda; simonanguma@gmail.com; 4 School of Public Health, Rutgers University, Piscataway, NJ 08854, USA; mengqi@sph.rutgers.edu

**Keywords:** particulate pollution, PM_10_, ambient air, Uganda, urbanization

## Abstract

Long-term particulate matter (PM_10_) measurements were conducted during the period January 2016 to September 2017 at three sites in Uganda (Mbarara, Kyebando, and Rubindi) representing a wide range of urbanization. Spatial, temporal and diurnal variations are assessed in this paper. Particulate matter (PM_10_) samples were collected for 24-h periods on PTFE filters using a calibrated pump and analyzed gravimetrically to determine the average density. Particulate levels were monitored simultaneously using a light scattering instrument to acquire real time data from which diurnal variations were assessed. The PM_10_ levels averaged over the sampling period at Mbarara, Kyebando, and Rubindi were 5.8, 8.4, and 6.5 times higher than the WHO annual air quality guideline of 20 µg·m^−3^, and values exceeded the 24-h mean PM_10_ guideline of 50 µg·m^−3^ on 83, 100, and 86% of the sampling days. Higher concentrations were observed during dry seasons at all sites. Seasonal differences were statistically significant at Rubindi and Kyebando. Bimodal peaks were observed in the diurnal analysis with higher morning peaks at Mbarara and Kyebando, which points to the impact of traffic sources, while the higher evening peak at Rubindi points to the influence of dust suspension, roadside cooking and open-air waste burning. Long-term measurement showed unhealthy ambient air in all three locations tested in Uganda, with significant spatial and seasonal differences.

## 1. Introduction

Numerous human and environmental health concerns are concomitant to poor air quality. Approximately 4.2 million deaths worldwide in 2016 occurred due to ambient air pollution [1]. Numerous health problems are associated with exposure to inhalable particulate matter (PM_10_, with aerodynamic diameter less than 10 micrometers) [2,3,4,5,6,7]. Although the consequences of poor air quality can be dire and deterioration is evident, no long-term air quality measurement has been reported in Uganda. However, a few short-term studies have reported polluted ambient air and recommended long-term measurements [8,9,10,11]. 

Uganda is a developing country in East Africa with a population of approximately 38 million people [12]. Its capital, Kampala, is highly urbanized, and numerous additional urban and sub-urban centers are emerging throughout the country. Most arise by the agglomeration of people due to necessity rather than planning, resulting in uncoordinated settlement and development [13]. For instance, in many of these urban and sub-urban centers, small- and large-scale factories are constructed side by side with residential housing. Also, these urban centers have many characteristics that have been linked to particulate generation, including open-air waste burning, vehicle traffic, biomass combustion, unpaved or poorly maintained roads, and open, loose surfaces on agricultural land and grass-free areas [10,14,15,16,17]. 

Numerous previous studies have reported high levels of particle pollution in cities across Africa [16,18,19,20,21,22]. In addition, temporal (seasonal, day of week, and diurnal) and spatial (small scale and synoptic) variations in PM levels have been reported, which have been attributed to differences in local source contributions, as well as local and synoptic weather variations [14,23,24,25,26]. However, no studies in Uganda have assessed temporal variations, and only two have reported spatial variations [8,11]. This lack of information may hinder abatement. 

Therefore, this study reports PM_10_ levels in Uganda sampled over a period of 20 months at three sites. The sites were selected to represent different levels of urban development in Uganda and hence reflect different settlement and land use patterns. PM_10_ was measured because it includes both coarse particles (2.5–10 μm in aerodynamic diameter) and fine particles (less than 2.5 μm). While the WHO attributes the majority of long-term morbidity and mortality effects to PM_2.5_ concentrations, short-term exposures to high PM_10_ levels are also a significant risk factor for mortality [13]. Additionally, PM_10_ includes re-suspended dust, which is likely to be an important contributor to particulate matter in Uganda. Spatial, seasonal, and diurnal variation in particle mass concentration were evaluated. 

## 2. Materials and Methods 

### 2.1. Study Area and Sampling Sites

**Figure 1:**
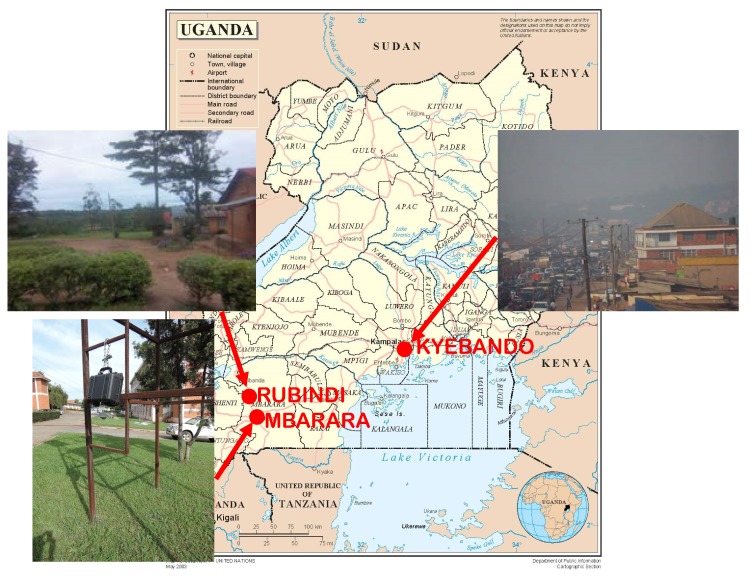
Data collection sites. The figure is based on the UN map of Uganda [28].

Samples were collected at the trading center of Rubindi, Mbarara University of Science and Technology (MUST) in Mbarara District, and Kyebando in Kampala, as shown in Figure 1. Sampling sites were located at (latitude, longitude): Mbarara (0.6148° S, 30.6551° E); Rubindi (0.32557° S, 30.57624° E); and Kyebando (0.36190° N, 32.57464° E). All three sites experience a bimodal climate (two dry and two wet seasons annually). Usually, dry seasons are during the months of December to January and June to August, while wet seasons are from February to May and September to November. At all sites, biomass in the form of either charcoal or wood is the major source of energy for cooking. Lighting is provided by a mixture of kerosene lamps and electricity from the national grid, solar PVs, and generators. Transportation is mainly by two-stroke motorcycles and old reconditioned vehicles imported from Asia on poorly maintained or unpaved roads. Open-air waste burning is a major method of waste management. 

Mbarara municipality is a fast-growing urban center in southwestern Uganda approximately 280 km from the capital of Kampala. Typical of developing urban centers in Uganda, Mbarara is experiencing rapid population growth, increasing traffic, a high rate of road, residential and commercial construction, and an increasing number of processing plants and factories. Roads are a combination of paved and unpaved. Sampling was done at Mbarara University of Science and Technology (town campus), which is approximately 2 km from Mbarara town center. The site was approximately 100 m from the Mbarara–Kabale road and approximately 50 m from the university canteens, in close proximity to the university library. The main sources of particulates are likely to be dust re-suspension from roads and fields, school cooking, open-air waste burning, and vehicular exhaust.

Kyebando is in the Mpererwe district of Kampala, the commercial and political capital of Uganda. Kampala has experienced enormous industrial and population growth in the last two decades. The demand for transportation, housing, energy, and waste management has also increased [27]. Due to these increasing demands, the number of vehicles (mostly reconditioned second-hand vehicles from Asia), road construction and maintenance activities, commercial, residential, and industrial building construction, open-air waste burning, and biomass burning have also increased, and these activities contribute to particulate pollution. The sampling site was in the back yard of a home approximately 500 m from Kalerwe market and 50 m from the Mpererwe–Gayaza road. The residential setting at the sampling site is a mixture of upscale and slum settlement. Evening open-air roadside cooking occurs along the Mpererwe–Gayaza road. 

Rubindi is a rural trading center approximately 40 km from Mbarara town along the Mbarara–Ibanda road. Typical of sub-urban centers in Uganda, Rubindi is characterized by an increasing population, small-scale agricultural processing factories, crowded settlements, and unpaved roads. Usually, open-air roadside cooking occurs in the evenings. In the surrounding villages, subsistence agriculture is the main economic activity. This study site is of mixed land use with no clear residential, commercial or industrial areas. Due to security reasons, sampling was done at St. Andrews Secondary School. The site was approximately 50 m from the Mbarara-Ibanda road, 20 m from an unpaved road, and 50 m from the school kitchen. The main sources of particulates are likely to be dust re-suspension from roads and fields, roadside and school cooking, and open-air waste burning.

### 2.2. Sample Collection and Preparation

Samples were collected from 4 January 2016, to 3 September 2017. Due to equipment limitations, samples were collected consecutively at different sites. At each site, sampling was conducted for 24-h periods on 3 to 5 days, after which the equipment was moved to the next site. Additionally, five filter blanks were obtained over the course of the sampling period, using the same procedure as for sample collection, but with a HEPA filter attached before the PEM inlet. These blank results are included in the data table in the supplementary materials (Table S1).

Samples were collected using a Personal Environmental Monitor (PEM, Model 200, TSI Inc., Shoreview, Minnesota, USA) designed to collect particles of aerodynamic diameter less than 10 µm on pre-weighed 37-mm PTFE filters using a Leland Legacy pump (SKC, Inc., Eighty Four, PA, USA). This instrument has been found by other researchers to be comparable to stationary samplers for measuring PM_10_ [29]. Simultaneous measurements were made with a real-time monitor (SidePak, Model AM510, TSI, Inc.) using an impactor with a 10-µm cut-off. Instruments were prepared for sampling in accordance with the instrument manuals [30,31]. A calibrator (Model 510-H, Mesa Laboratories, Inc., Lakewood, CO, USA) was used to set the pumping speed to 10 liters/min for the Leland Legacy and 1.7 liters/min for the SidePak. The Leland Legacy pump rate was remeasured after each twenty-four-hour sampling period. Pumps were packaged in a waterproof plastic box with inlets connected using short tubes to points outside the box sheltered from rain and hung at approximately 1.5 m above the ground, as shown in Figure 1 for the Mbarara site. External batteries were used to allow the pump power to extend for 24 hours. Samples collected on filters were packaged in petri dishes and shipped to Rutgers University for post-sample weighing. 

Pre- and post-collection filter weighing was conducted following the US Environmental Protection Agency (EPA) protocol in an EPA-audited laboratory at Rutgers University. Filters were equilibrated under a temperature- and relative humidity-controlled environment for 48 hours (21.5 ± 1.5 °C, 35 ± 5% RH) prior to weighing. Filter weights were then determined using a microbalance digital scale (Cahn C-30, Cahn Instruments, Cerritos, CA, USA; or Mettler MT5, Mettler Toledo, Columbus, OH, USA). Each filter was weighed twice, and the overall coefficient of variation for repeated filter measurements was less than 1%.

### 2.3. Data Preparation

For the filter-based measurements described in Section 3.1, Section 3.2 and Section 3.3, the 24-h mean concentrations were computed from the filter mass gain and the volume of air pumped over the 24-h period. The pump rates at the beginning and the end of the sampling period were averaged when calculating the volume of air pumped through the filter. Using notes taken during sampling, valid samples were selected. Because of the relatively large number of samples collected, the sample means could be calculated with more certainty than is implied by the standard deviation of the sample population. The reported standard deviations of the means are calculated using *σ*_mean_ = *σ*_pop_/$\sqrt{N}$; that is, the uncertainty in the mean is smaller than the population standard deviation by the square root of *N*, the number of data points. For the real-time light-scattering measurements described in Section 3.4, the SidePak real-time sampler recorded the one-minute average mass concentrations from which the hourly averages were determined. 

### 2.4. Ethical Approval

This study was exempted from ethical review by the Mbarara University of Science and Technology research ethics committee. Research approval was granted by the Uganda National Council of Science and Technology under registration number PS 37. 

## 3. Results

### 3.1. Spatial Variations in Air Quality

A summary of descriptive statistics is presented in Table 1. All samples were collected for 24-h periods. While it was not possible to collect samples simultaneously at different sites, the spread of sampling dates over a period of 20 months and the number of samples collected at each site (~30) enables statistical analysis of site differences without concern that the samples at one site might be all be either high or low due to a single weather pattern. In fact, the high degree of day-to-day variation observed (see Table S1 in the Supplementary Materials) suggests that much of the particulate matter is due to local sources such as open-air waste burning and therefore would not be correlated between locations even if measured on the same day. 

Sample means were approximately 5.8, 8.4, and 6.5 times higher than the WHO annual PM_10_ air quality guideline (20 µg·m^−3^) and 1.7, 2.4, and 1.9 times higher than the WHO interim target guideline (70 µg·m^−3^) at Mbarara, Kyebando, and Rubindi, respectively. Statistically significant differences among the sample means were observed (one-way ANOVA, *F* = 3.227 and *p* = 0.045). Pairwise Tukey’s test comparisons between Mbarara and Rubindi, Mbarara and Kyebando, and Kyebando and Rubindi yielded *p*-values of 0.758, 0.041 and 0.191, respectively, implying that the differences in the PM_10_ mass concentrations were statistically significant only between Mbarara and Kyebando. 

The WHO 24-h PM_10_ air quality guideline (50 µg·m^−3^) was exceeded 83, 100, and 86% of the time at Mbarara, Kyebando, and Rubindi, respectively, while the WHO interim target 1 (150 µg·m^−3^) was exceeded 23, 66, and 36% of the time. For the one-sample t-test using the annual air quality guideline and interim target 1, *p*-values < 0.05 were obtained at all sites, implying that both the annual standards were exceeded at all sites. A summary of the test results is in Table S2 of the Supplementary Materials. 

### 3.2. Seasonal Differences in Air Quality

**Figure 2:**
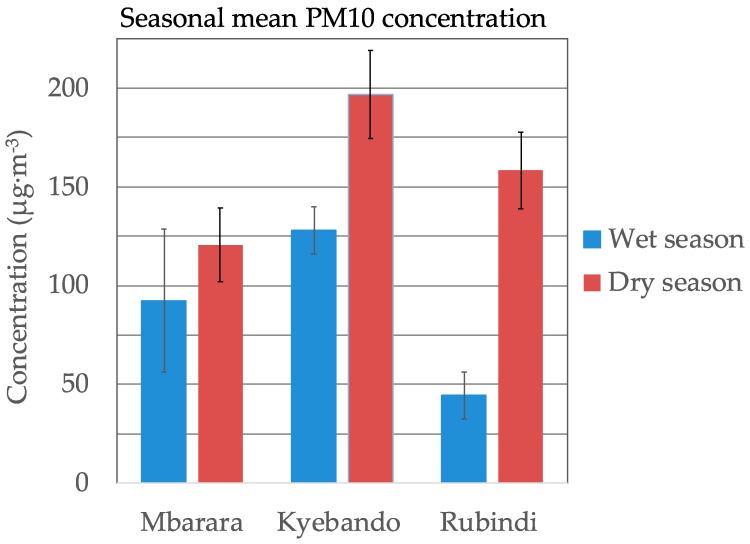
Comparison of mean PM_10_ during dry and wet seasons. Error bars represent one standard deviation of the mean.

A summary of the seasonal mean PM_10_ levels is presented in Figure 2. The wet and dry seasons were determined by the calendar (wet seasons are from February to May and September to November), with small adjustments if rain persisted into a nominally dry period or had not yet fallen at the beginning of a wet period. At all sites, higher means were observed during dry seasons than wet seasons. However, the magnitudes of seasonal differences were not similar at all sites. The *p*-value obtained using the independent sample t-test was >0.05 at Mbarara and <0.05 at Kyebando and Rubindi, implying statistically significant seasonal differences at Kyebando and Rubindi but not at Mbarara. A summary of t-tests is presented in Table S3 in the Supplementary Materials.

### 3.3. Weekday/Weekend Differences in Air Quality

**Figure 3:**
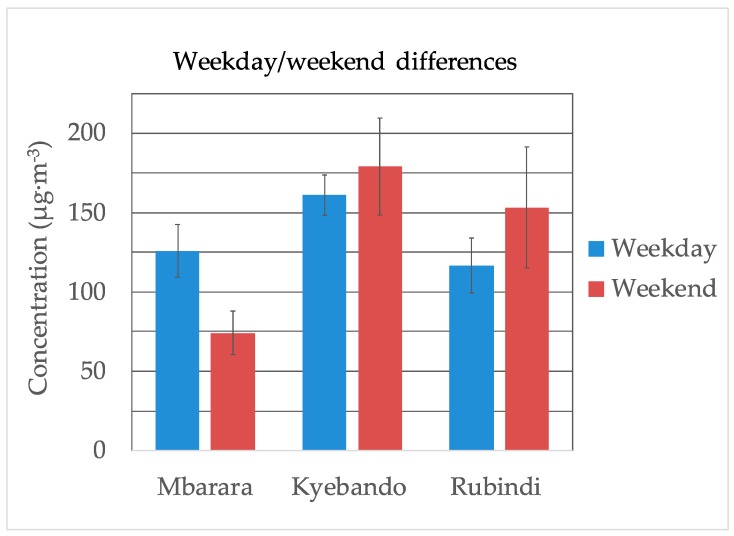
Comparison of weekday/weekend mean PM_10_ concentration. Error bars represent one standard deviation of the mean.

A summary of the weekday/weekend mean PM_10_ concentration is presented in Figure 3. The mean PM_10_ concentration was higher during the weekend than during weekdays at Rubindi and Kyebando and lower at Mbarara. Using the independent samples t-test, *p*-values > 0.05 were obtained at Rubindi and Kyebando and a *p*-value < 0.05 was obtained at Mbarara. This implied that at 95% confidence interval, there were no statistically significant differences between weekday/weekend PM_10_ levels at Kyebando and Rubindi and a statistically significant difference at Mbarara. A summary of the t-test is in Table S3 in the Supplementary Materials.

### 3.4. Diurnal Variations

**Figure 4:**
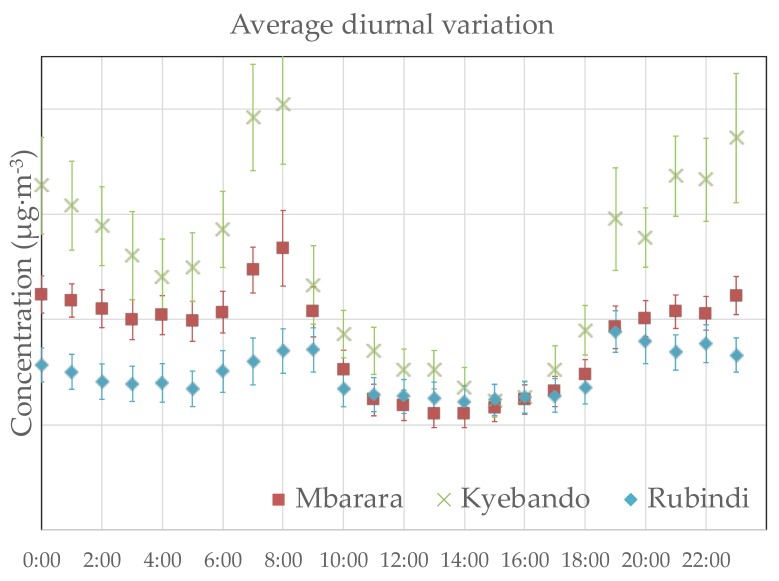
Diurnal variations in particulate matter concentration. These data were collected using the real-time instrument. Readings for each day were averaged by the hour, and the same hour was averaged over all sampling days to yield the plotted points (*N* = 39, 25, 30 for Mbarara, Kyebando, and Rubindi). Error bars are the standard deviation of the mean for the same hour on all sampling days. See Table 3 for a comparison of gravimetric and real-time concentration measurements.

Average diurnal variations at Mbarara, Rubindi, and Kyebando are presented in Figure 4.

Bimodal peaks were observed at all sites between 06:00 to 09:00 and 19:00 to 23:00. At all sites, low values were observed from approximately 10:00 to approximately 18:00, after which the concentration started to increase to a maximum that occurred between 19:00 and 23:00 hours. A slow drop was observed after 23:00 to approximately 05:00. After 05:00, a gradual increase was observed peaking at approximately 08:00 and dropping to a minimum at approximately 15:00. At Mbarara and Kyebando, the morning peak time averages were slightly greater than evening peak time averages, while at Rubindi, the evening peak time average was slightly greater than the morning peak time average.

Diurnal concentration variations in PM_10_ have implications for air pollution exposure. We have stratified the measured PM_10_ levels by daytime (10:00 to 18:00) vs. overnight (19:00 to 09:00). These averages are shown in Table 2, along with results from the independent samples t-test. Overnight concentrations were significantly higher than daytime for all locations. High PM_10_ levels at nighttime are likely caused by temperature inversion or different source contributions. In future studies, we will further explore the diurnal variation in PM_10_ species concentrations and associated sources. 

Table 3 shows concentrations measured by the SidePak in comparison to gravimetric measurements. Averaging over all the samples collected, concentrations measured by the SidePak were 70% of concentrations measured by gravimetric methods. The SidePak instrument is factory calibrated to report mass densities based on the light-scattering properties of the respirable fraction of standard ISO 12103-1, A1 Test Dust (formerly Arizona Test Dust). The calibration is dependent on the size distribution and material properties of the particulates, which may not have the same average properties as the standard dust, and also may not have constant properties over time or location in Uganda, resulting in readings that differ from direct measurements of the mass.

## 4. Discussion

### 4.1. Air Quality and Spatial Differences

Both short- and long-term analysis revealed poor ambient air quality at all sites, even those that are less urbanized. In high-income countries, particulate levels are typically higher in urban areas than in areas with lower population densities. The assumption that the same is true in East Africa may be one reason why most previous studies in this region have concentrated on urban areas. The high levels of particulates measured in the Kyebando region of Kampala, PM_10_ = 167 ± 13 µg·m^−3^, were similar to those found in other studies. A previous study measured PM_10_ on two days in the Mpererwe region of Kampala and found PM_10_ levels of 133 µg·m^−3^ and 208 µg·m^−3^ [10]. 

In this study, the PM_10_ levels found in other regions were nearly as high: 130 ± 17 µg·m^−3^ in Rubindi and 116 ± 14 µg·m^−3^ in Mbarara. Levels of particulates in less densely populated areas have previously been measured in only two regions of Uganda and a few regions of East Africa. Values of PM_2.5_ in rural Uganda were measured to average 31.4 and 20.2 µg·m^−3^, considerably lower than the values found in urban areas [11,32]. Similarly, measurements on Mt. Kenya found PM_2.5_ averaging 6.9 ± 1.3 µg·m^−3^ compared to the daytime average of 50–100 µg·m^−3^ in Nairobi [17]. Only one research team has reported similar values between urban and rural locations: measurements in a rural site in Tanzania found dry and wet season PM_10_ levels of 61 and 48 µg·m^−3^ [24], while the same technique yielded values of 76 and 52 µg·m^−3^ in Dar el Salaam [15]. However, these levels are much lower than those found in this study. 

It is particularly striking in this study that the mean PM_10_ levels were higher (although not reaching the level of significance) in Rubindi than in Mbarara, since normally an urban area would be assumed to have higher particulate levels than a rural area. This variation points to the importance of local conditions on PM levels. The levels in Mbarara may have been lower because the site was on a university campus, well separated from residential neighborhoods and businesses, with a higher fraction of grass and pavement, and with less open-air waste burning or cooking than at the other two sites. In contrast, at Rubindi, higher particle concentrations could have resulted due to significant mineral dust suspension from loose open surfaces, open-air waste burning, evening roadside cooking, and biomass combustion, all of which occurred at close proximity to the sampling site. 

### 4.2. Seasonal Differences

Seasonal differences in PM_10_ concentration were statistically significant at Rubindi and Kyebando, but not Mbarara. We have not found other studies of seasonal variations in Uganda, but they have been observed in other areas of East Africa. As stated above, Mkoma et al. measured PM_2.5_ levels that varied somewhat between dry and wet seasons [15]. Similarly, Gaita et al. measured seasonal variation in Nairobi, with PM_2.5_ averaging 25 µg·m^−3^ in the dry season and 8.9 µg·m^−3^ in the wet season [17]. The lower overall levels at Mbarara and the decreased seasonal variation suggest that the factors that would be expected to cause seasonal variations (mineral dust suspension, open-air waste burning and agricultural burning) may be present at lower levels at the Mbarara site than other two sites, due to its location on a university campus. 

### 4.3. Weekday/Weekend Differences

Although differences in weekday/weekend PM_10_ levels were observed at all sites, the differences were not statistically significant at Rubindi and Kyebando. Other studies that have reported differences in the weekday/weekend PM concentrations [33,34,35,36] showed that pollution levels are lower during weekends than weekdays due to the decline in traffic activities. The observation in this study suggests that the differences in traffic activities between weekends and weekdays are not significant in Rubindi and Kyebando but may be significant in Mbarara. In Uganda, most businesses and schools operate 6–7 days per week, so the differences between traffic on weekends and weekdays are not very noticeable, although, on the MUST campus in Mbarara, most classes are taught on weekdays. This decreased level of activity on weekends may be responsible for the lower weekend particulate concentrations observed.

### 4.4. Diurnal Variations

Bimodal peaks were observed at all sites. Higher morning than evening peaks were observed at Mbarara and Kyebando, while a higher evening than morning peak was observed at Rubindi. Similar observations reported for other locations [37,38,39] were attributed to increased particle emission during morning and evening hours due to increased traffic and variations in the planetary boundary layer. Higher morning peak time averages at Kyebando and Mbarara point to the influence of morning time traffic, while the higher evening peak time average at Rubindi suggests that dust suspension, roadside cooking, and open-air waste burning may play a significant role in escalating particle pollution. In all locations, significantly higher concentrations were observed during the overnight hours (19:00–09:00) compared to the daytime hours (10:00–18:00).

## 5. Conclusions

In this study, PM_10_ mass concentration was monitored in Uganda at three sites that represent different levels of urban development. While this was a limited sampling in terms of both the number of sites and the number of samples collected at each, it was the first study of particulates in Uganda across seasons or using real-time instruments, enabling the reporting of not only spatial, but also seasonal and diurnal variations. Significant spatial differences were observed, which points to differences in source emission characteristics. However, surprisingly, the trading center of Rubindi experienced higher mean concentrations than the small city of Mbarara (although not significantly higher) and the mean concentrations in both locations were less than 1/3 lower than in the large city of Kampala. Higher concentrations were observed during dry seasons than wet seasons, which may be due to the influence of seasonal sources such as dust suspension and open-air waste burning and weather. Significant weekday/weekend differences were observed only at Mbarara. The bimodal diurnal peaks that showed higher morning peak time than evening peak time at Kyebando and Mbarara may be due to the importance of early morning traffic, while higher evening peaks at Rubindi may be due to dust re-suspension, roadside cooking and open-air waste burning. 

The measurements also showed a surprising amount of variation from day to day, as can be seen in Table S1 of the supplementary material. This variation implies that local sources may be important contributors to particulate levels and points to the importance of studies that are both longer term and sample a variety of local conditions.

Despite these variations, measurements at all seasons and locations indicate that the WHO guidelines were exceeded. The levels of exceedance of the WHO guidelines that were observed at all sites has been associated with more than a 5 and 15% increase in short- and long-term mortality, respectively, making mitigation highly desirable [40]. Given the likelihood of different types of sources dominating in different levels of urbanization, mitigation may need to proceed differently in different locations. Future studies that examine the chemical composition of the samples collected may be able to determine the sources more definitively.

## Figures and Tables

**Table 1:** Daily particulate matter (PM_10_) concentrations (µg·m^−3^) measured at three different locations. *N* is the number of samples; SD is the standard deviation of the mean as described in Section 2.3; Min and Max are the minimum and maximum observed values. All samples were collected for 24 h.

Site	*N*	Mean ± SD	Min	25th Percentile	Median	75th Percentile	Max
Mbarara	30	116 ± 14	30	57	98	144	361
Kyebando	29	167 ± 13	50	124	167	184	399
Rubindi	28	130 ± 17	17	72	115	167	477

**Table 2:** Comparison of daytime/overnight PM_10_ levels (µg·m^−3^) measured at three different locations using light-scattering techniques. *N* is the number of samples for which valid light-scattering data were collected at each location.

Site	*N*	Mean Daytime (10:00 to 18:00)	Mean Overnight (19:00 to 09:00)	*t*-Value	*p*-Value
Mbarara	39	66	107	−9.007	0.000
Kyebando	25	77	151	−6.872	0.000
Rubindi	30	64	80	−5.603	0.000

**Table 3:** Comparison of PM_10_ levels (µg·m^−3^) measured at three different locations using gravimetric and light-scattering techniques. *N* is the number of samples for which both sets of data were available and SD is the standard deviation of the mean.

Site	*N*	Mean ± SD (Gravimetric)	Mean ± SD (Light Scattering)
Mbarara	29	116 ± 14	93 ± 10
Kyebando	20	173 ± 17	128 ± 12
Rubindi	24	136 ± 20	75 ± 9

## Supplementary Materials

The following are available online at https://www.mdpi.com/1660-4601/16/10/1752/s1, Table S1: Summary of data collection. The sampling locations are described in Section 2 of the paper. Each data collection period was 24 hours, starting at the date and time shown. Gravimetric samples were collected on filters in the PEM, while optical data were collected by the SidePak. Seasons were generally consistent with the expected dates (dry = December–January and June–August; wet = February–May and September–November), but some samples near the transitions were re-assigned based on observed rainfall. Weekends are Saturdays and Sundays. For sampling times that overlap weekdays and weekends, such as those starting in the late afternoon on a Friday or Sunday, the assignment was made based on the majority of the sampling time. Missing data are left blank, Table S2: Summary of the one sample t-test for the 24-h means in the three sampling locations. The annual air quality guideline and interim target values were used as standard means, Table S3: Summary of the independent sample t-test comparing seasonal and day of week differences. The means for each data set are shown along with the standard deviation of the mean. *t*-(*p*-) values are the values of statistical test parameters *t*- value and *p*-value (probability).
