# Supplementary material for: Spatio-Temporal Variation in the Concentration of Inhalable Particulate Matter (PM10) in Uganda
Source: Int J Environ Res Public Health. 2019 May 17;16(10):1752. doi: 10.3390/ijerph16101752 (PMC6571861; doi:10.3390/ijerph16101752)
Supplement: Supplementary file 1 [file ijerph-16-01752-s001.pdf]

Supplementary material

Table S1. Summary of data collection. The sampling locations are described in Section 2 of the paper. Each data collection period was 24 hours, starting at the date and time shown. Gravimetric samples were collected on filters in the PEM, while optical data were collected by the SidePak. Seasons were generally consistent with the expected dates (dry = December-January and June-August; wet = February-May and September-November), but some samples near the transitions were re-assigned based on observed rainfall. Weekends are Saturdays and Sundays. For sampling times that overlap weekdays and weekends, such as those starting in the late afternoon on a Friday or Sunday, the assignment was made based on the majority of the sampling time. Missing data are left blank.

Location	Start date	Start time	Gravimetric (µg m^{-3})	Optical (µgm^{-3})	Season	Weekend/weekday
Mbarara	4-Jan-16	16:05	172	70	Mixed	Weekday
Mbarara	6-Jan-16	11:55	104		Mixed	Weekday
Mbarara	8-Jan-16	10:40	143	104	Mixed	Weekday
Mbarara	10-Jan-16	9:04	137	139	Mixed	Weekend
Mbarara	11-Jan-16	17:20	93	113	Mixed	Weekday
Rubindi	27-Jan-16	9:32	231	136	Dry	Weekday
Rubindi	28-Jan-16	17:56	240	122	Dry	Weekday
Rubindi	30-Jan-16	8:23	167	173	Dry	Weekend
Rubindi	2-Feb-16	8:31	200	144	Dry	Weekday
Blank	3-Feb-16	11:18	46			
Mbarara	29-Mar-16	10:57	153	76	Wet	Weekday
Mbarara	30-Mar-16	11:40	287	78	Wet	Weekday
Rubindi	2-Apr-16	17:00	83	14	Wet	Weekend
Kyebando	2-Jun-16	16:30	194	225	Mixed	Weekday
Kyebando	6-Jun-16	12:10	173	258	Mixed	Weekday
Kyebando	9-Jun-16	12:20	194	160	Mixed	Weekday
Rubindi	13-Jun-16	13:20	190	62	Dry	Weekday
Rubindi	14-Jun-16	14:20	162	76	Dry	Weekday
Rubindi	15-Jun-16	15:15	111	86	Dry	Weekday
Rubindi	16-Jun-16	16:15	116	70	Dry	Weekday
Mbarara	22-Jun-16	16:18	186	94	Dry	Weekday
Mbarara	23-Jun-16	16:36	147	85	Dry	Weekday
Mbarara	24-Jun-16	17:10	77	97	Dry	Weekend
Mbarara	25-Jun-16	18:05	56	95	Dry	Weekend
Mbarara	26-Jun-16	18:55	54	109	Dry	Weekday
Blank	27-Jun-16	19:05	-7			
Kyebando	2-Jul-16	12:53	399	141	Dry	Weekend
Kyebando	5-Jul-16	10:16	191	141	Dry	Weekday
Kyebando	7-Jul-16	7:05	52	100	Dry	Weekday
Kyebando	9-Jul-16		193	186	Dry	Weekend
Rubindi	15-Jul-16	15:20	477	94	Dry	Weekend
Rubindi	16-Jul-16	16:15	110	91	Dry	Weekend
Rubindi	17-Jul-16	16:50	73	69	Dry	Weekday
Rubindi	18-Jul-16	17:35	66	101	Dry	Weekday
Rubindi	19-Jul-16	18:20	132	109	Dry	Weekday
Mbarara	1-Aug-16	13:14	361	208	Dry	Weekday
Mbarara	2-Aug-16	14:21	156	220	Dry	Weekday
Mbarara	3-Aug-16	15:02	58	159	Dry	Weekday
Mbarara	4-Aug-16	15:45	99	182	Dry	Weekday
Mbarara	5-Aug-16	16:30	78	168	Dry	Weekend
Kyebando	8-Aug-16	10:35	285		Dry	Weekday
Kyebando	10-Aug-16	10:43	167		Dry	Weekday
Blank	15-Aug-16	11:30	12			

Kyebando	9-Nov-16	2:47	158		Wet	Weekday
Kyebando	10-Nov-16	3:45	178		Wet	Weekday
Kyebando	13-Nov-16	10:32	50		Wet	Weekend
Kyebando	15-Nov-16	11:33	102		Wet	Weekday
Kyebando	17-Nov-16	10:49	147		Wet	Weekday
Rubindi	5-Dec-16	10:38	154	0	Dry	Weekday
Rubindi	6-Dec-16	12:46	64	0	Dry	Weekday
Blank	7-Dec-16	14:15	3			
Mbarara	25-Jan-17		251	88	Dry	Weekday
Mbarara	26-Jan-17		96	74	Dry	Weekday
Mbarara	28-Jan-17		44	25	Dry	Weekend
Mbarara	30-Jan-17		84	61	Dry	Weekday
Kyebando	17-Feb-17	14:22	155		Wet	Weekend
Kyebando	20-Feb-17	11:18	138		Wet	Weekday
Mbarara	1-May-17	9:25	30	32	Wet	Weekday
Mbarara	2-May-17	12:40	35	33	Wet	Weekday
Rubindi	4-May-17	9:30	17	16	Wet	Weekday
Rubindi	5-May-17	11:10	17	17	Wet	Weekday
Rubindi	7-May-17	15:05	22	17	Wet	Weekday
Rubindi	8-May-17	16:25	23	13	Wet	Weekday
Mbarara	10-May-17	9:30	43	33	Wet	Weekday
Mbarara	11-May-17	12:10	46	47	Wet	Weekday
Mbarara	12-May-17	13:30	53	44	Wet	Weekend
Blank	21-May-17	9:15	16			
Kyebando	14-May-17	13:30	97	71	Wet	Weekend
Kyebando	15-May-17	14:40	121	71	Wet	Weekday
Kyebando	16-May-17	3:40	128	112	Wet	Weekday
Kyebando	17-May-17	16:55	74	123	Wet	Weekday
Kyebando	31-May-17	11:30	188	79	Wet	Weekday
Kyebando	2-Jun-17	11:30	150	81	Dry	Weekday
Kyebando	4-Jun-17	11:50	179	152	Dry	Weekend
Kyebando	5-Jun-17	13:00	167	123	Dry	Weekday
Mbarara	9-Jun-17	8:40	102	75	Dry	Weekday
Mbarara	19-Jun-17	7:40	109	59	Dry	Weekday
Mbarara	20-Jun-17	9:00	84	47	Dry	Weekday
Mbarara	21-Jun-17	10:15	129	80	Dry	Weekday
Rubindi	22-Jun-17	13:40	167	64	Dry	Weekday
Rubindi	23-Jun-17	14:50	109	68	Dry	Weekend
Rubindi	24-Jun-17	17:15	115	25	Dry	Weekend
Rubindi	21-Jul-17	12:26	202	69	Dry	Weekend
Rubindi	22-Jul-17	13:10	122	65	Dry	Weekend
Rubindi	23-Jul-17	15:25	117	101	Dry	Weekday
Kyebando	3-Aug-17	12:40	254	150	Dry	Weekday
Kyebando	4-Aug-17	14:25	240	152	Dry	Weekend
Kyebando	5-Aug-17	13:40	206	21	Dry	Weekend
Kyebando	26-Aug-17	13:30	175	106	Dry	Weekend
Kyebando	27-Aug-17	14:10	96	99	Dry	Weekend
Rubindi	1-Sep-17	13:16	83	201	Wet	Weekend
Rubindi	2-Sep-17	14:40	66	244	Wet	Weekend

Table S2. Summary of the one sample t-test for the 24-hour means in the three sampling locations. The annual air quality guideline and interim target values were used as standard means.

Site	Air quality guideline 20 μg m^{-3} *t*-value (*p*-value)	Interim target one 70 μg m^{-3} *t*-value (*p*-value)
Mbarara	6.806 (0.000)	3.245 (0.003)
Kyebando	11.184 (0.000)	7.387 (0.000)
Rubindi	6.278 (0.000)	3.421 (0.002)

Table S3. Summary of the independent sample t-test comparing seasonal and day of week differences. The means for each data set are shown along with the standard deviation of the mean. *t-* (*p-*) values are the values of statistical test parameters t- value and p-value (probability).

	Mbarara		Kyebando		Rubindi	
	**dry**	**wet**	**dry**	**wet**	**dry**	**wet**
number of samples	18	7	14	12	21	7
mean	121 ± 19	92 ± 36	197 ± 22	128 ± 12	158 ± 19	44 ± 12
t-value (dry-wet)	0753		2.595		3.293	
p-value (dry-wet)	0.229		0.008		0.001	
	weekday	weekend	weekday	weekend	weekday	weekend
number of samples	24	6	19	10	18	10
mean	126	74	161	179	117	153
t-value (weekday-end)	1.506		−0.639		−1.003	
p-value (weekday-end)	0.072		0.264		0.163	
